# Supplementary material for: Barbed arrow-like structure membrane with ultra-high rectification coefficient enables ultra-fast, highly-sensitive lateral-flow assay of cTnI
Source: Nat Commun. 2024 Jul 3;15:5603. doi: 10.1038/s41467-024-49810-z (PMC11222510; doi:10.1038/s41467-024-49810-z)
Supplement: Supplementary file 1 — Supplementary Information [file 41467_2024_49810_MOESM1_ESM.pdf]

Supplementary Information for:

Barbed arrow-like structure membrane with ultra-high rectification coefficient enables ultra-fast, highly-sensitive lateral-flow assay of cTnI

Author list

Juanhua Li[1, 2, †], Yiren Liu[1, 2, †], Tianyu Wu[1, 2], Zihan Xiao[1, 2], Jianhang Du[3], Hongrui Liang[1, 2], Cuiping Zhou[4], Jianhua Zhou[1, 2, *]

[†] These authors contributed equally.

Affiliations

[1] School of Biomedical Engineering, Shenzhen Campus of Sun Yat-sen University, Shenzhen, Guangdong 518107, China

Juanhua Li, Yiren Liu, Tianyu Wu, Zihan Xiao, Hongrui Liang, Jianhua Zhou.

[2] Key Laboratory of Sensing Technology and Biomedical Instruments of Guangdong Province, School of Biomedical Engineering, Sun Yat-sen University, Guangzhou 510275, China;

Juanhua Li, Yiren Liu, Tianyu Wu, Zihan Xiao, Hongrui Liang, Jianhua Zhou.

[3] Guangdong Innovative Engineering and Technology Research Center for Assisted Circulation, the Eighth Affiliated Hospital of Sun Yat-sen University, Shenzhen 518033, China

Jianhang Du.

[4] Department of Emergency, Nanfang Hospital, Southern Medical University, Guangzhou, Guangdong 510515, China

Cuiping Zhou.

Contents

Supplementary Discussion 1 | The principle of unidirectional flow of water on BAS Mem.
Supplementary Discussion 2 | The condition for unidirectional flow of water on BAS Mem.
Supplementary Discussion 3 | Formula calculation showing there was sufficient time for the immunological binding between "antigen-antibody-nanogold" complex and the antibody immobilized at the T line, when sample solution flow through the T line.

Supplementary Notes 1 | High processing and preservation costs of fiber-based chromatographic membranes.
Supplementary Notes 2 | Advantages of cellulose acetate in the preparation of lateral-flow strips.

Supplementary Methods 1 | The definition of the parameter of barbed-arrow like structure, and the design concept of the BAS Mem.
Supplementary Methods 2 | The composition of the hydrophilic coating used for surface treatment of the BAS Mem, highlighting its good bonding capabilities and wear resistance, as well as the methods for adjusting the hydrophilicity of BAS Mem through the chemical modification of hydrophilic coating.

Supplementary Fig. 1 | Schematic drawings show three analyses of forces generated by the side walls when liquid flowed backwards to the sharp corners on membranes modified with different contact angles.
Supplementary Fig. 2 | Schematic drawing shows the decomposition of the force generated by the sides in the coordinate system.
Supplementary Fig. 3 | Parameters of a structural unit on BAS Mem.
Supplementary Fig. 4 | SEM images of membranes with different structural parameters.
Supplementary Fig. 5 | Histograms indicate the effect of different parameters of BAS Mem on lengths of liquid spreading in forward and backward direction and rectification coefficient, k.
Supplementary Fig. 6 | Plots indicate the effect of different parameters of BAS Mem on velocities of liquid flowing on the membranes.
Supplementary Fig. 7 | Microstructure images of BAS Mem fabricated with different materials.
Supplementary Fig. 8 | Characterization of residues of F-BSA on BAS Mem and NC Mem.
Supplementary Fig. 9 | SEM images exhibit different batches of membranes.
Supplementary Fig. 10 | Optical images exhibit the time-dependent flow of liquid on different batches of BAS Mem.
Supplementary Fig. 11 | The hydrophilicity of BAS Mem that stored for various durations.

Supplementary Fig. 12 | The simulate results of unidirectional flow of a droplet on BAS Mem.
Supplementary Fig. 13 | The simulate results show the flow of a droplet on membranes with different surface contact angles.
Supplementary Fig. 14 | Histogram shows the average flow velocities of liquid on BAS Mem and NC Mem.
Supplementary Fig. 15 | Radar plot shows the performance of BAS Mem when it was used as the component of lateral-flow strips.
Supplementary Fig. 16 | Results of lateral flow strips constructed with BAS Mem and NC Mem for the assay of positive sample solution.
Supplementary Fig. 17 | The Q-Q plot exhibits the results for mimic samples taken from Figure 4.
Supplementary Fig. 18 | Images show the original results of 25 serum samples tested by lateral-flow strips.
Supplementary Fig. 19 | Characterization of flow velocity of F-BSA solution at the T line, and on the lateral-flow strip.
Supplementary Fig. 20 | Characterization of the shape, microstructure, and pore size distribution of CA bands.
Supplementary Fig. 21 | The schematics shows the time is sufficient for the immunological binding between the "antigen- antibody- nanogold" complex and the antibody immobilized at the T line.
Supplementary Fig. 22 | Results of lateral flow strips constructed with BAS Mem and NC Mem for the assay of positive sample solution using different antibody-labeled signal amplification nanoprobe.
Supplementary Fig. 23 | Schematic diagram shows the design concept of the BAS Mem.
Supplementary Fig. 24 | Schematic graphs show the steps for preparing the hydrophilized BAS Mem.
Supplementary Fig. 25 | Characterization of the lateral-flow strip made of BAS Mem.

Supplementary Table 1 | Table exhibits the parameters of membranes with different angles, *φ*.
Supplementary Table 2 | Table exhibits the parameters of membranes with different heights, *H*.
Supplementary Table 3 | Table exhibits the parameters of membranes with different scaling factors, *W*.
Supplementary Table 4 | Table exhibits the parameters of membranes with different spacings, *S*.

Supplementary References

Supplementary Discussion 1 | The principle of unidirectional flow of water on BAS Mem.

1. Forces generated by the side walls as water flows backwards

Supplementary Fig. 1 showed the force analysis of side walls based on the definition of Gibbs' inequality. We discussed the forces when water flows backwards to the sharp corners. In this case, the forces generated by the sidewalls, denoted as $\mathbf{F}_{r1}$, acting as the resistance to the backward flow of water. Here, δ is defined as the angle between the tangent line of meniscuses at the corner and the angle with the radial line.

As shown in Supplementary Fig. 1-A, when $0° < \theta < \alpha$, $\delta \in \left(0, \ \frac{\pi}{2} - \alpha + \theta\right)$. There has the expression as:

$$\mathbf{F}_{r1} = \sigma \Delta h \sin \delta \quad (1)$$

As shown in Supplementary Fig. 1-B, when $\theta = \alpha$, $\delta = \frac{\pi}{2}$. There has the expression as:

$$\mathbf{F}_{r1} = \sigma \Delta h \quad (2)$$

As shown in Supplementary Fig. 1-C, when $\alpha < \theta < 90°$, $\delta \in \left(0, \ \frac{\pi}{2} - \alpha + \theta\right)$. There has the expression as:

$$\mathbf{F}_{r1} = \sigma \Delta h \sin(\pi\text{-}\delta) = \sigma \Delta h \sin \delta \quad (3)$$

Therefore, one can write $\mathbf{F}_{r1}$ generalized for any θ as:

$$\mathbf{F}_{r1} = \sigma \Delta h \sin \delta \quad (4)$$

After substituting $\Delta h \in (0, \ H)$, $\delta \in \left(0, \ \frac{\pi}{2} - \alpha + \theta\right)$ into the equation (4). We got the expression of $\mathbf{F}_{r1}$ as:

$$\mathbf{F}_{r1} = \sigma \int_0^{H} \int_0^{\frac{\pi}{2}-\alpha+\theta} \Delta h \sin \delta \, d\Delta h \, d\delta \quad (5)$$

$$\mathbf{F}_{r1} = \frac{\sigma H^2}{2} [1 - \sin(\alpha - \theta)] \quad (6)$$

2. Forces generated by the undersides as water flows backwards

We also studied the forces generated on the underside when water flows. It always the driving force for the flow of water. l represented the projected length of the flowing meniscuses on the x-axis (Supplementary Fig. 2-A). In consequence, forces along the y-axis generated by undersides, denoted as F_{ud}, could be written as (Supplementary Fig. 2-B):

$$\mathbf{F}_{ud} = \int_0^{l} \sigma \cos \theta \, dl \quad (7)$$

$$\mathbf{F}_{ud} = \sigma l \cos \theta \quad (8)$$

In view of $l = W$ when water flows backwards to the sharp corners. Therefore, forces generated by undersides when water flow backwards, $\mathbf{F}_{ud1}$, could be written as:

$$\mathbf{F}_{ud1} = \sigma W \cos \theta \quad (9)$$

3. Forces generated by the side walls as water flows foreward

Supplementary Fig. 2-C showed the analysis of the forces generated by the side walls when water flows forward, so that $\mathbf{F}_s$, generated by side walls, could be written as:

$$\mathbf{F}_s = \sigma \frac{H}{\sin\theta} \tag{10}$$

γ is the angle between the tangent plane of side wall and the x-y plane, with $\gamma \in (0, \beta)$.

$\mathbf{F}_1$ was defined as the component force which perpendicular to the tangent plane, while $\mathbf{F}_2$ was parallel to that. Then $\mathbf{F}_1$ and $\mathbf{F}_2$ were written respectively as:

$$\mathbf{F}_1 = \mathbf{F}_s \sin\theta = \sigma H \tag{11}$$

$$\mathbf{F}_2 = \mathbf{F}_s \cos\theta \sin\theta = \sigma H \cos\theta \tag{12}$$

Therefore, the driving force generated by side walls, $\mathbf{F}_{sd2}$, expressed as:

$$\mathbf{F}_{sd2} = \mathbf{F}_2 \cos\gamma = \sigma H \cos\theta \cos\gamma \tag{13}$$

The resistance generated by side walls, $\mathbf{F}_{r2}$, expressed as

$$\mathbf{F}_{r2} = \mathbf{F}_1 \sin\gamma = \sigma H \sin\gamma \tag{14}$$

4. Forces generated by the underwalls as water flows foreward

As for the forward case, it was still a driving force which generated by underside. Thus, $\mathbf{F}_{ud2}$ could be written as follow with $l \in (W, D)$.

$$\mathbf{F}_{ud2} = \sigma l \cos\theta \tag{15}$$

Supplementary Discussion 2 | The condition for unidirectional flow of water on BAS Mem.

When the liquid was pinned on the BAS Mem, the total force acting on the meniscus should satisfy $\mathbf{F}_{\mathrm{tp}} > 0$, which expressed as:

$$\sigma H^2[1 - \sin(\alpha - \theta)] - \sigma W \cos\theta > 0 \qquad (16)$$

We substituted H=0.32 mm, W=0.16 mm, α=45° into the equation (16) and got：

$$0.32^2 \times \left[1 - \frac{\sqrt{2}}{2}(\cos\theta - \sin\theta)\right] > 0.16\cos\theta$$

$$1.45\cos\theta - 0.45\sin\theta < 0.64$$

$$\theta > 48°$$

When the liquid flow forward on the BAS Mem, the total force acting on the meniscus should satisfy $\mathbf{F}_{\mathrm{td}} > 0$, which expressed as:

$$\sigma l\cos\theta + 2\sigma H\cos\theta\cos\gamma - 2\sigma H\sin\gamma > 0 \qquad (17)$$

$$\cos\theta > \frac{1}{\frac{l}{2H\sin\gamma} + \cot\gamma} \qquad (18)$$

In order for the above equation to hold, one should take the maximum value of γ and minimum value of l, which were denoted as γ_{max} and l_{min}. We substituted H =0.32 mm, $\gamma_{max} = \beta$ =30°, $l_{min} = W$ =0.16 mm into the equation (18) and got：

$$\cos\theta > \frac{1}{\frac{1}{2\times 0.32\sin 30°} + \cot 30°}$$

$$\cos\theta > 0.448$$

$$\theta < 64°$$

In summary, the theoretical calculation condition for the unidirectional flow of liquid on the BAS Mem was 48°< θ < 64°.

Supplementary Discussion 3 | Formula calculation showing there was sufficient time for the immunological binding between "antigen-antibody-nanogold" complex and the antibody immobilized at the T line, when sample solution flow through the T line.

The immunological binding between the "antigen-antibody-nanogold" complex and the antibody in T line was primarily influenced by the time taken for the free diffusion of "antigen-antibody-nanogold" complex to the antibody immobilized in at the T line (which was fixed to the surface of the CA fiber). According to Fick's law and the Einstein equation, we got the equation (19):

$$<x^2>=6DT \tag{19}$$

Herein, x represented the distance of free diffusion of sample molecules or nanoprobe (such as "antigen-antibody-nanogold" complex), D was the diffusion coefficient of "antigen-antibody-nanogold" complex, and T was the time of free diffusion of "antigen-antibody-nanogold" complex. We approximately set the distance of free diffusion of sample molecules as 2.605 μm (which was the half of the average pore size of CA band), according to the Supplementary Fig. 20 C.

Therefore, we determined that it took approximately 0.25 s for the "antigen-antibody-nanogold" complex to freely diffuse to the immobilized antibody in the T line through the calculation of the above equation. Considering it took 8.00 s for the sample solution carrying the target sample molecules to pass through the T line (Supplementary Fig. 19). Consequently, there is sufficient time for the "antigen-antibody-nanogold" complex to immunological binding with the antibody in T line at the current flow velocity of liquid (Supplementary Fig. 21).

Supplementary Notes 1 | High processing and preservation costs of fiber-based chromatographic membranes.

Existing fiber-based chromatographic films typically use cellulose derivatives as raw materials. In the production of the chromatographic membrane, the cellulose derivative containing slurry needs to be spread over a large area of the planar carrier and the solvent of the slurry evaporated by air drying to obtain the formed film. It is necessary to control the drying temperature and air blast speed at various places throughout the planar carrier during this process to ensure that the solvent of the cellulose derivative slurry evaporates at the same rate at each place to form fiber filaments of uniform size and pore. As a result, it is difficult to produce lamellar membranes of consistent quality, which makes them more expensive to process.

In addition, they require more stringent protection to maintain the good quality of the chromatography membrane during long-term storage, owing to the cellulose derivatives are flammable. For example, cellulose nitrate, which is most commonly used in constructing LFA strips, is also used in the production of explosive mixtures. Cellulose derivatives are also susceptible to decomposition or oxidation into shorter fragments by light or airborne oxidants during long-term storage, which leads to brittleness and discoloration of the fibrous membranes, resulting in a deterioration of their quality. Therefore, storage conditions need to be carefully controlled to maintain the quality of the cellulose membranes during long-term storage, which raises storage costs.

Supplementary Notes 2 | Advantages of cellulose acetate in the preparation of lateral-flow strips.

Firstly, this CA material is easily fabricated into porous fibrous structures, providing enough space for the immobilization of antigens and antibodies, and meets the requirements for constructing T and C lines in LFA.

Secondly, the chemical structure and surface properties of CA make it less likely to non-specifically adsorb the antigens and antibodies, which helps to reduce background noise, and enhancing the SNR of the LFA.

Additionally, CA material performs good biocompatibility, chemical stability, and durability, making it widely suitable for various biological applications, particularly due to its strong tolerance and stability against changes in pH values and different types of solvents.

Finally, the ease of processing CA material allows CA could be fabricated into products of various shapes and sizes through conventional processing techniques easily (such as extrusion, injection molding, and blow molding) at a relatively low cost.

In summary, material of CA provides multiple advantages in the preparation of T and C lines, which meet the requirements of the proposed lateral-flow strips.

Supplementary Methods 1 | The definition of the parameter of barbed-arrow like structure, and the design concept of the BAS Mem.

1. The definition of the parameter of barbed-arrow like structure.

In order to determine the parameters of the groove structure, we referred to several structural parameters mentioned in the classical theoretical formulations. These structural parameters could affect the forces generated by sidewalls of the structure on the liquid. And we further combined them with the needs of practical applications for LFA.

In our work, we primarily use the sharp edges on the sidewalls of microchannels to prevent the wetting and spreading of liquid, and making use of the surface tension of liquid and capillary forces to drive the liquid, thereby achieving unidirectional liquid transport. Therefore, in designing the barbed arrow-like structure (BAS), we paid special attention to the shape and size parameters of the BAS sidewalls. We adjusted the shape of the BAS sidewalls through four key variables: α represents the arc degree of the short arc sidewall, β represents the arc degree of the long arc sidewall. W represents the shortest width between the long arc sidewalls within the structural unit, and H represents the height of the sidewalls. In selecting these four parameters, we primarily considered several key factors including fluid dynamics, surface tension balance, and the interaction between fluid and structure.

We designed and optimized the arc degree of the short arc sidewall (α), and the arc degree of the long arc sidewall (β) under the guidance of the Gibbs inequality. And we determined the width (W) and height (H) of microchannels, guided by the flow behavior of liquid in square capillary microchannels. Additionally, considering the practical application requirements and the specific demands for the components of lateral-flow strip, we also included the spacing between each row of channels into our design parameters for detailed definition, represented by the letter S. The details about the guideline are listed as follows:

(1) The Gibbs inequality

The classical Gibbs inequality is not only very important in the study related to liquid surface tension and contact angle, but also has significant implications when discussing the wetting and spreading behavior of liquids on solid surfaces. The Gibbs inequality can be expressed as equation (20):

$$\theta - \theta_0 \leq 180° - \varphi \tag{20}$$

Where θ is the actual contact angle of liquid on the surface, θ_0 is the equilibrium contact angle of the liquid, and φ is the angle of the sharp edge of sidewall. The Gibbs inequality reveals that whether a liquid, upon contacting a sharp edge (such as the edge of a microcavity), can be pinned (i.e., no longer continue to spread or slide) depends on its contact angle and the geometric shape of the edge. If this inequality is satisfied, then the liquid will be pinned at that sharp edge and cannot continue to spread beyond the edge. Thus, we know that in addition to the contact angle, the geometric shape of the edge is also an important factor affecting liquid behavior. Therefore, we chose to adjust the sharp corners of the BAS structure sidewalls as a parameter to modulate fluid behavior. We believe that by adjusting the angle of the sidewalls, we can control the behavior of liquids on the BAS Mem, effectively achieving liquid pinning. This discovery is significant for optimizing the BAS Mem structure and enhancing its performance in controlling liquid flow.

(2) Many researchers have conducted comprehensive analyses of liquid flow behavior in classical square capillary microchannels. For instance, Ichikawa et al. derived a formula

for capillary force in square capillary microchannels[1]. Their research indicates that the cross-sectional dimensions of the channel, namely width and height, significantly influence the capillary flow of liquid. Therefore, we selected the spacing (W) between the sidewalls and the height (H) of the sidewalls as two key parameters for adjusting the shape of the BAS structure.

2. The design concept of the BAS Mem.

The design concept of the BAS Mem is shown in Supplementary Fig. 23. Initially, we noted the foundation that liquid could achieve capillary flow through capillary force in the square capillary microchannel membranes. According to the research by Ichikawa et al., the parameters of H and W could affect the capillary force, so that they were the key parameters of capillary flow. However, this type of surface structure membrane has limitations in directional transport and efficiency, and resulting in ordinary performance in flow velocity. Therefore, we introduced symmetrical triangular prism blocks into the microchannels. The height of these blocks was the same as the depth of the square capillary microchannel. The addition of these blocks changed the geometric shape of the sidewalls, which also resulted in the change of region for liquid flow. When the liquid flowed through the blocks, it could be found that the angle (φ) between the neighboring two sidewalls of the blocks could primarily influence flow behavior of liquid. By adjusting φ to a small acute angle, we created the sharp edges on the channel sidewalls. According to the Gibbs inequality, we could make it difficult for the liquid to wet the sharp edges on the sidewalls in the backward flow direction, by adjusting the contact angle of surface of BAS Mem. Therefore, we prevented the reverse wetting and spreading of the liquid, and realized the unidirectional flow that facilitated by the sidewall. In further design improvement, we adjusted the structure and edge curvature of the prismatic blocks to create curved sidewalls. This design could reduce dead zones of fluid, turbulence, and flow resistance, thus enhancing the efficiency of liquid flow. All these design improvements aimed to enhance the performance of the BAS Mem in microfluidic applications, and ensuring the transport efficiency and directionality flow of liquid.

Supplementary Methods 2 | The composition of the hydrophilic coating used for surface treatment of the BAS Mem, highlighting its good bonding capabilities and wear resistance, as well as the methods for adjusting the hydrophilicity of BAS Mem through the chemical modification of hydrophilic coating.

The main component of the hydrophilic coating we used in this study, was the prepolymer of acrylic acid resin. The hydrophilic coating contained a sufficient number of hydrophilic functional groups. The hydrophilic coating could bond well to the substrate. Besides, the hydrophilic coating also exhibited good wear resistance after being coated on the surface of the BAS Mem.

Also, the surface hydrophilicity of BAS Mem was adjusted by the composition of hydrophilic coatings used in practical applications. We could effectively control the hydrophilicity and hydrophobicity of the hydrophilic coating by adjusting the length and flexibility of molecular chain, molecular weight of the resin, as well as the types and proportions of additives, and thereby regulating the surface hydrophilicity of the BAS Mem.

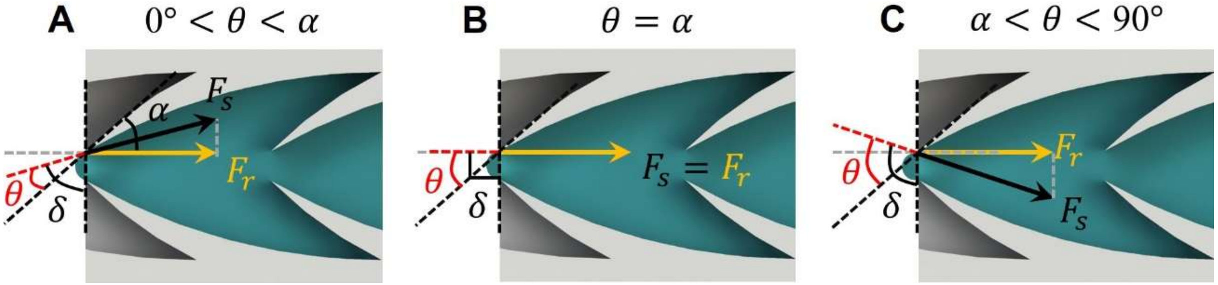

Supplementary Fig. 1 | Schematic drawings show three analyses of forces generated by the side walls when liquid flowed backwards to the sharp corners on membranes modified with different contact angles. (**A**) When $0° < \theta < \alpha$. (**B**) When $\theta = \alpha$. (**C**) When $\alpha < \theta < 90°$.

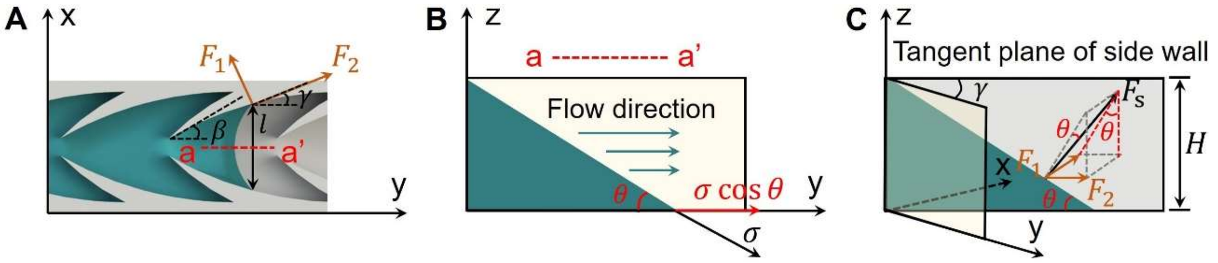

Supplementary Fig. 2 | Schematic shows the decomposition of the force generated by the sides in the coordinate system. (**A**) Schematic drawing exhibits flow behavior of water on the membrane in the x-y plane. (**B**) Schematic drawing exhibits decomposition of the force along the y-axis generated by the underside in the z-y plane. (**C**) Schematic drawing of the forces generated by the side wall as the water flows forward. Here, γ denoted the angle between the tangent plane of the side wall (gray) and the z-y plane (yellow).

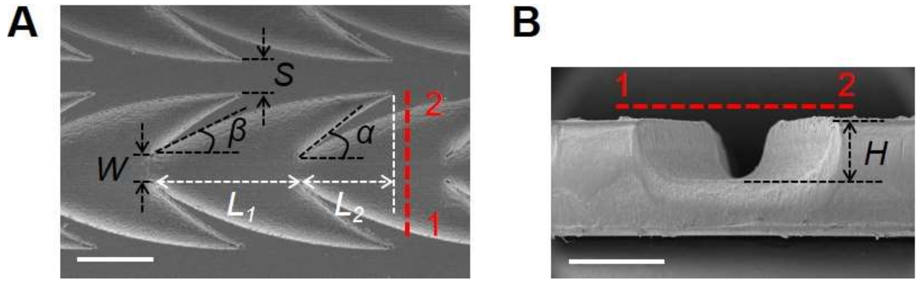

Supplementary Fig. 3 | Parameters of a structural unit on BAS Mem. (**A**) Top-view of BAS Mem with structural parameters. Here, the parameters marked with black letters were the variables we defined. α denoted the degrees of the short arc on the short-arc sidewalls, and β denoted the degrees of the long arc on the long-arc sidewalls. W denoted the shortest width between the long-arc sidewalls within a structure unit. S denoted the minimum spacings between neighboring microchannels. The parameters marked with white letters had: $L_1 = 6.25W$， $L_2 = 3.75W$. Scale bar= 500 μm. (**B**) Cross-sectional view of BAS Mem with structural parameters. H denoted the heights of the sidewalls. Scale bar= 500 μm.

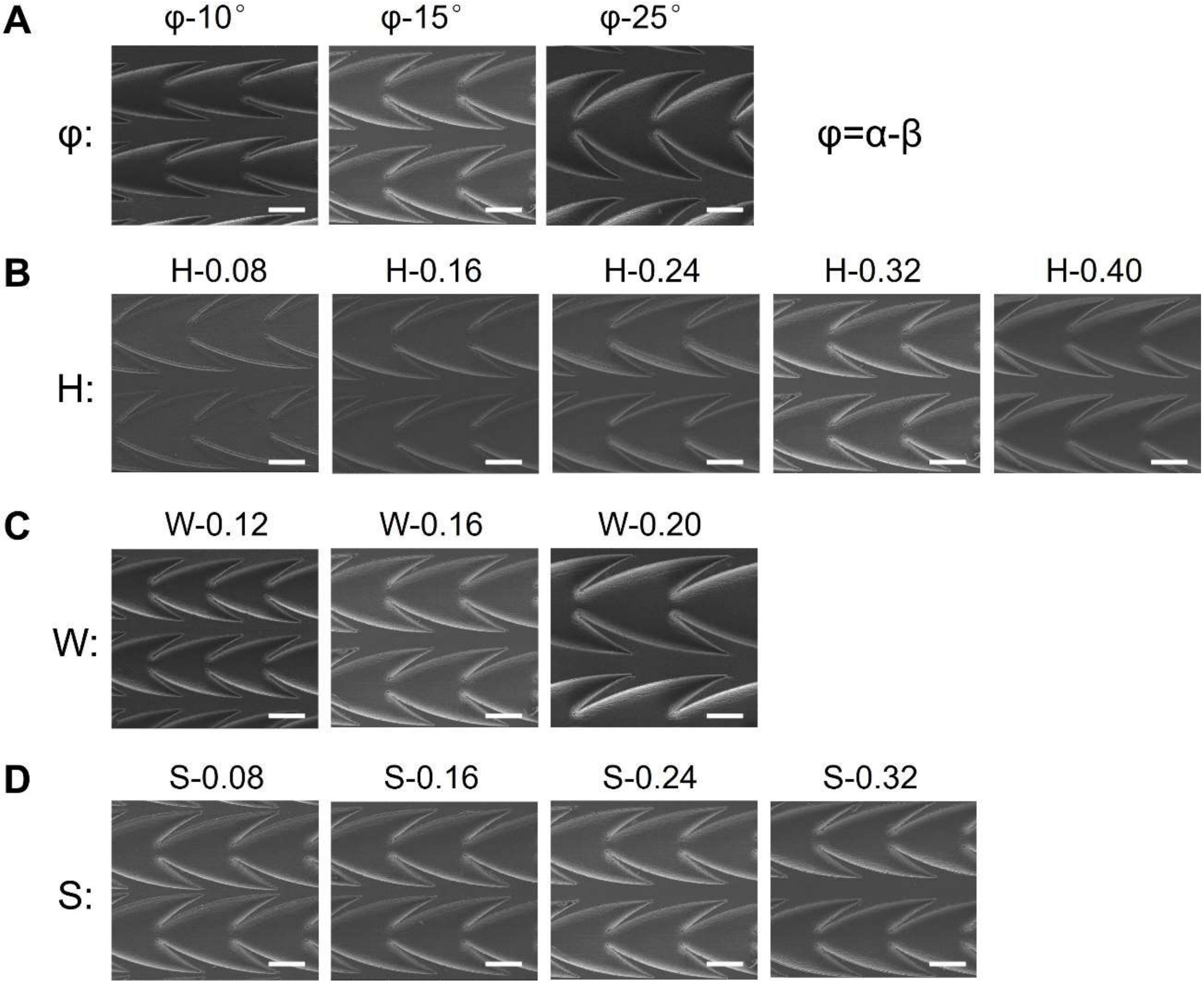

Supplementary Fig. 4 | SEM images of membranes with different structural parameters. Scale bar= 500 μm. (**A**) Membranes with different angles, φ. Here, φ=α-β. (**B**) Membranes with different heights, H. (**C**) Membranes with different scaling factors, W. (**D**) Membranes with different spacings, S.

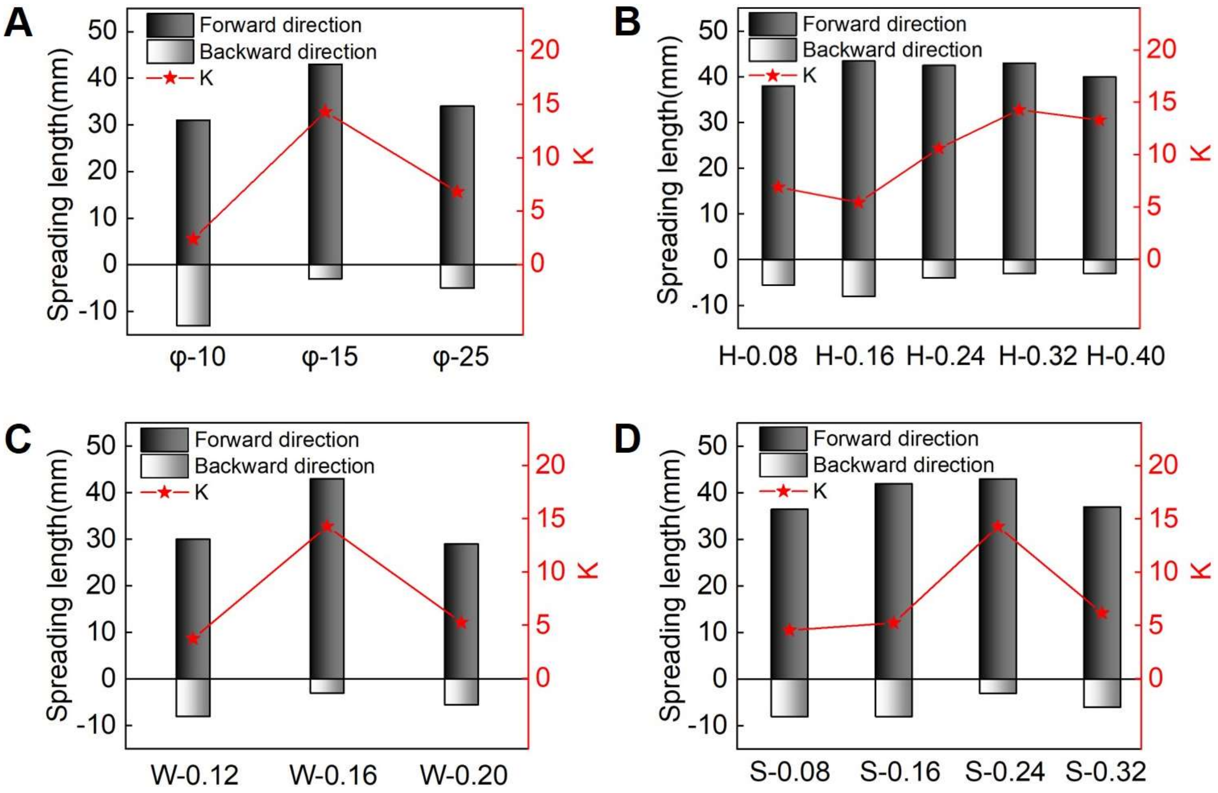

Supplementary Fig. 5 | Histograms indicate the effect of different parameters of BAS Mem on lengths of liquid spreading in forward and backward direction and rectification coefficient, k. Parameters including(**A**) φ; (**B**) H; (**C**) W; (**D**) S. Here, φ=α-β.

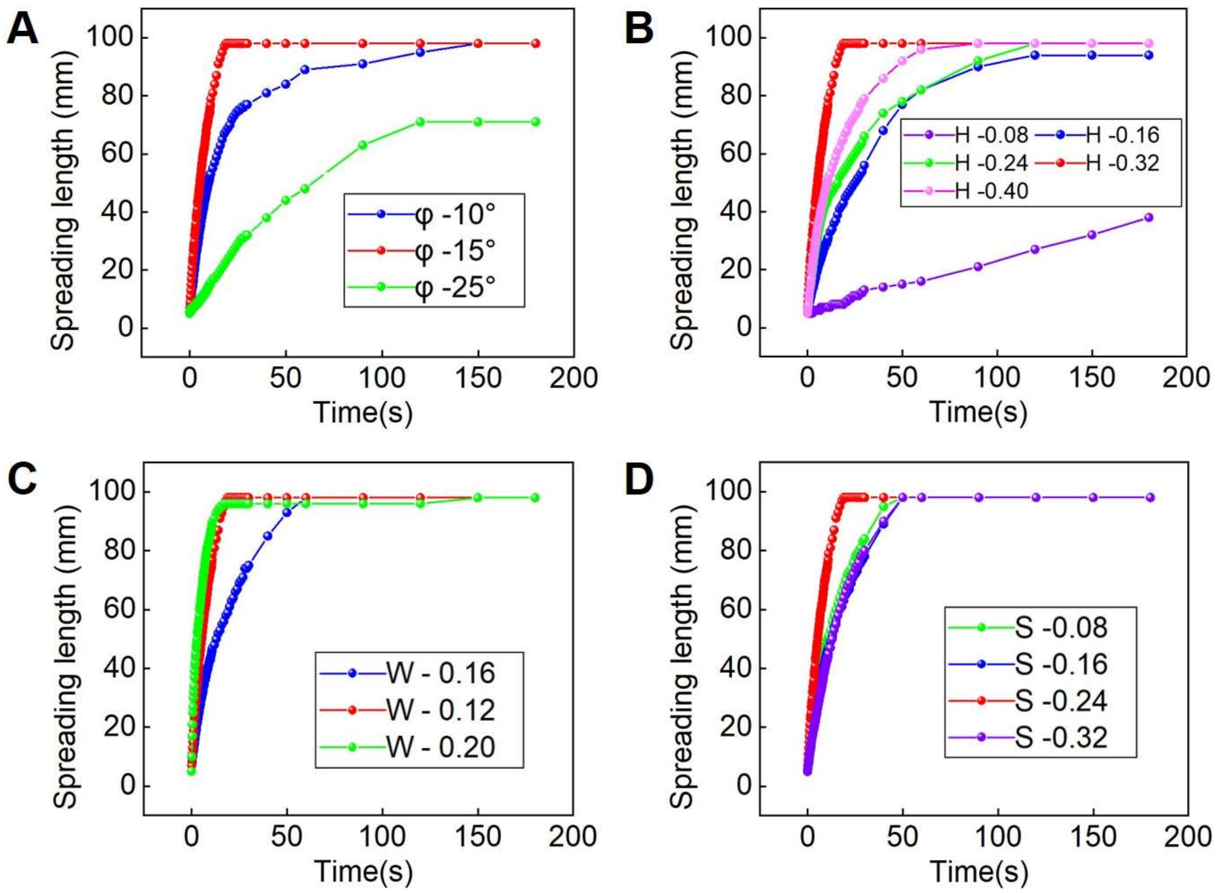

Supplementary Fig. 6 | Plots indicate the effect of different parameters of BAS Mem on velocities of liquid flowing on the membranes. Parameters including(**A**) φ; (**B**) H; (**C**) W; (**D**) S. Here, φ=α-β.

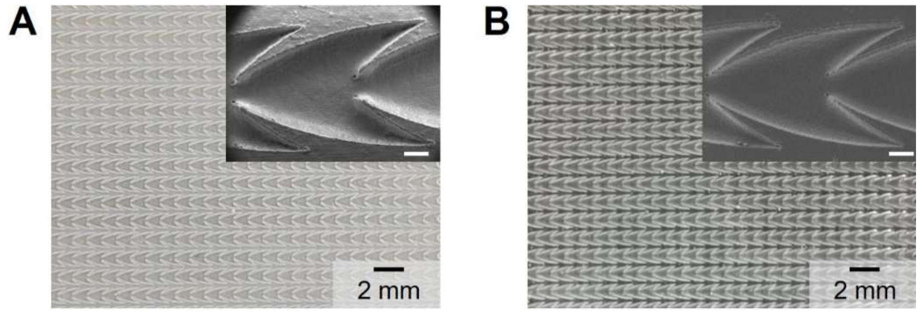

Supplementary Fig. 7 | Microstructure images of BAS Mem fabricated with different materials. Optical images of BAS Mem fabricated using substrates of (**A**) PP and (**B**) PMMA. The insets show the magnified SEM images respectively. Scale bar= 200 μm.

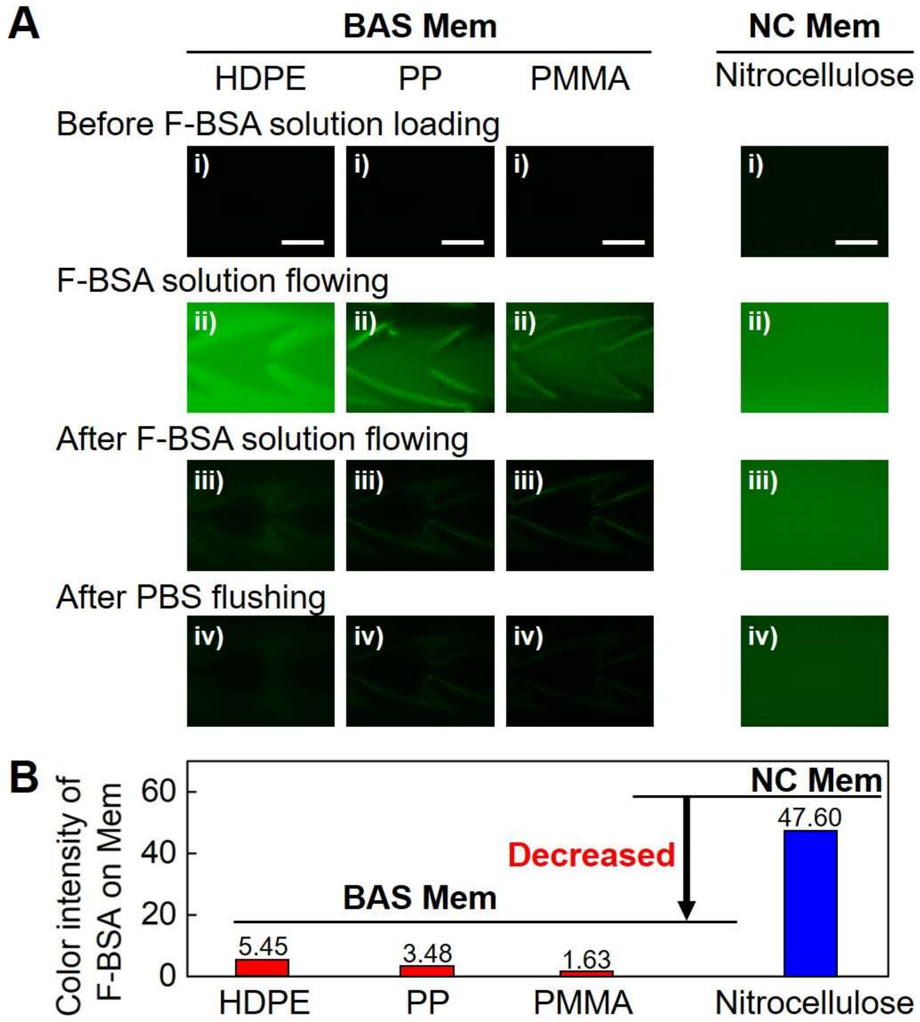

Supplementary Fig. 8 | Characterization of residues of F-BSA on BAS Mem and NC Mem. (**A**) Optical images showing the flow of F-BSA solution on BAS Mem and NC Mem, and the BAS Mem and NC Mem after flushing by PBS. Scale bar = 0.5 mm. (**B**) Comparative histogram shows the residual color intensity of F-BSA on BAS Mem and NC Mem after flushing by PBS.

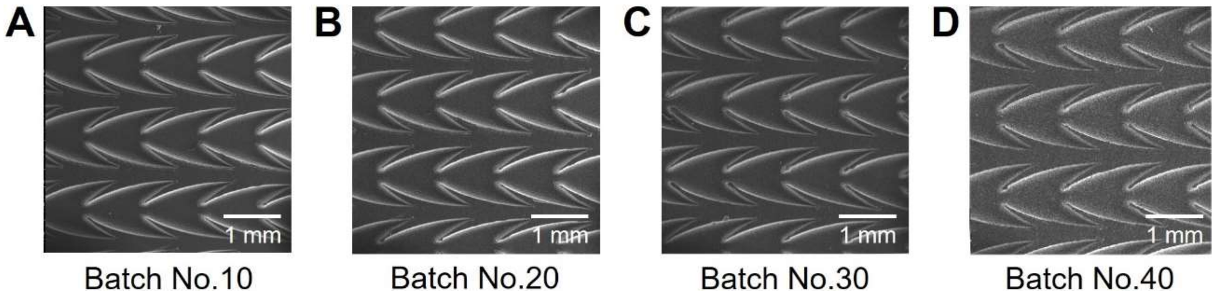

Supplementary Fig. 9 | SEM images exhibit different batches of membranes. (**A**) Tenth batch. (**B**) Twentieth batch. (**C**) Thirtieth batch. (**D**) Fortieth batch.

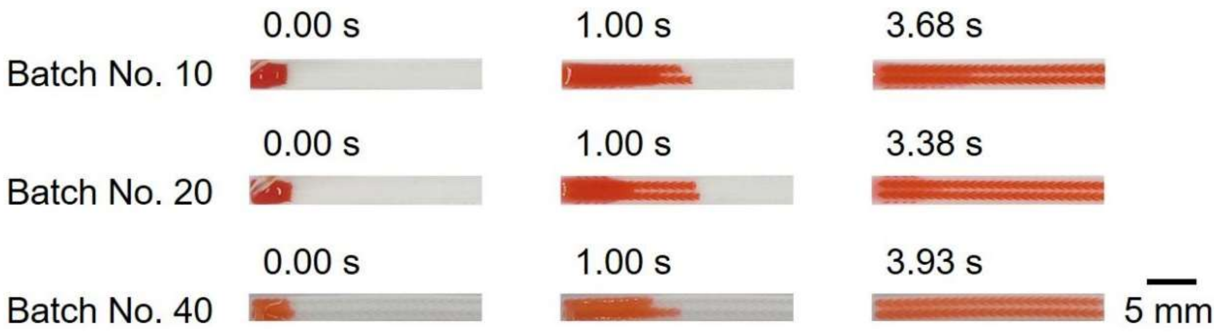

Supplementary Fig. 10 | Optical images exhibit the time-dependent flow of liquid on different batches of BAS Mem. Each batch of BAS Mem could carry liquid for 20.0 mm in 4.0 s.

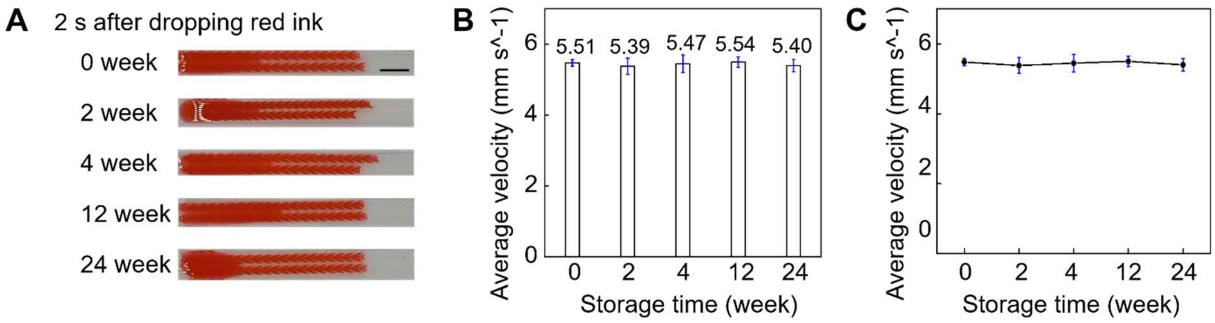

Supplementary Fig. 11 | The hydrophilicity of BAS Mem that stored for various durations. (**A**) Optical images showing the flow behavior of liquid on BAS Mem that stored for various durations. (**B**) Histogram showing the flow velocity of liquid on BAS Mem that stored for various durations. (**C**) The plot showing the relationship between the flow velocity of liquid on BAS Mem and the stored durations. **B** and **C** represent mean ± standard deviation (SD) of three independent experiments.

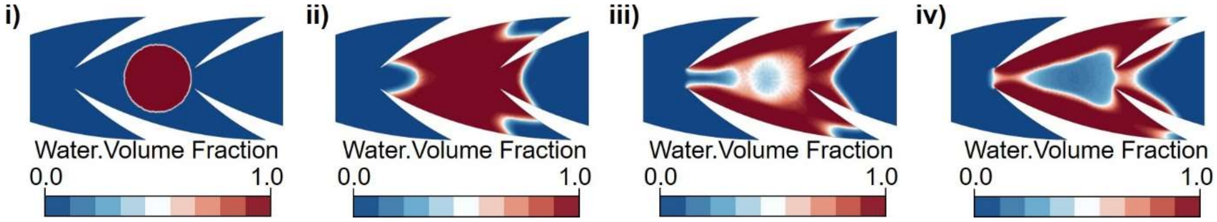

Supplementary Fig. 12 | The simulate results of unidirectional flow of a droplet on BAS Mem.

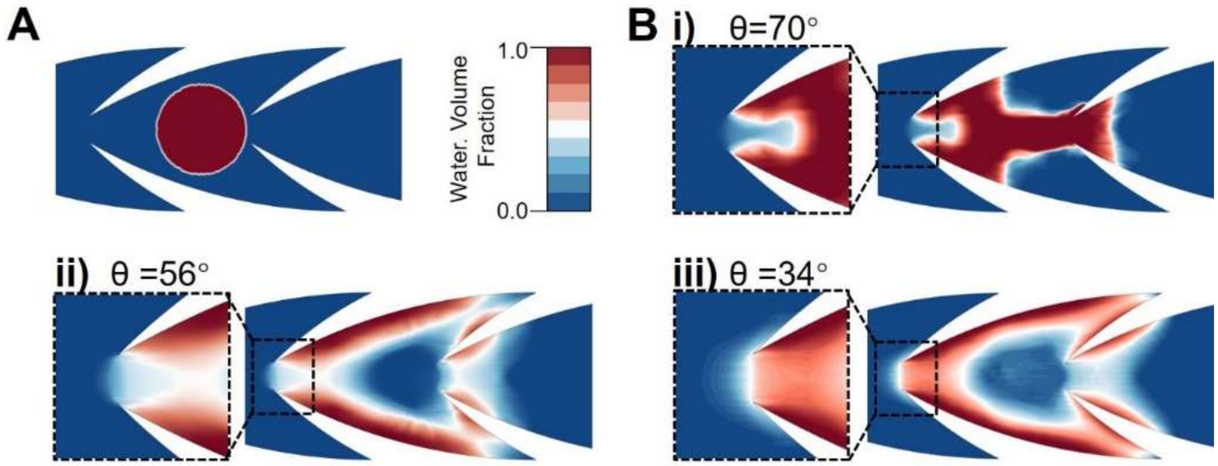

Supplementary Fig. 13 | The simulate contour diagrams show the flow of a droplet on membranes with different surface contact angles. (**A**) Image shows the droplet just deposited on BAS Mem. (**B**) Images show the droplet after spreading on membranes with different surface contact angles (θ).

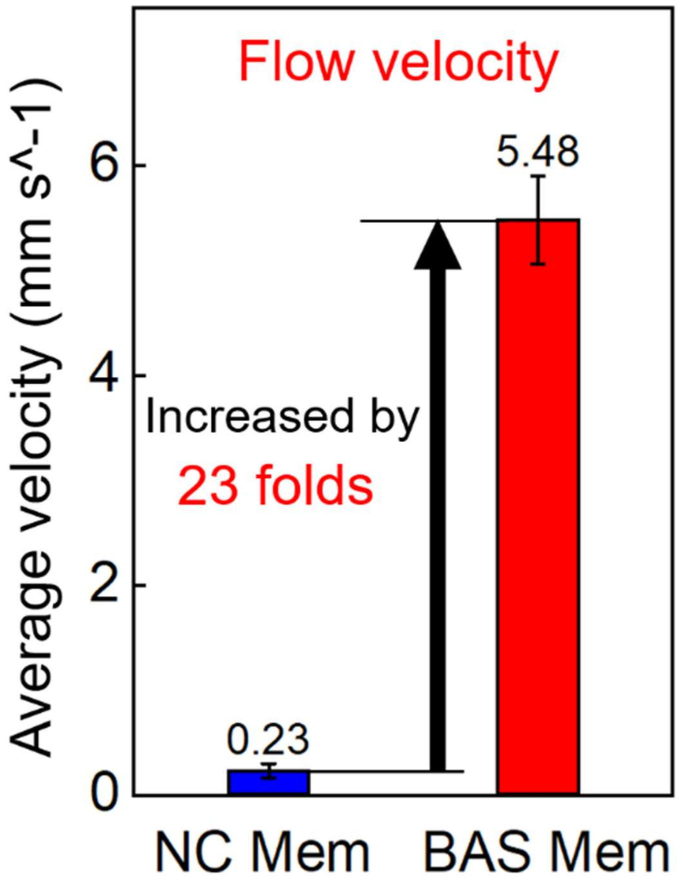

Supplementary Fig. 14 | Histogram shows the average flow velocities of liquid on BAS Mem and NC Mem. The bars represent mean ± SD of three independent experiments.

.

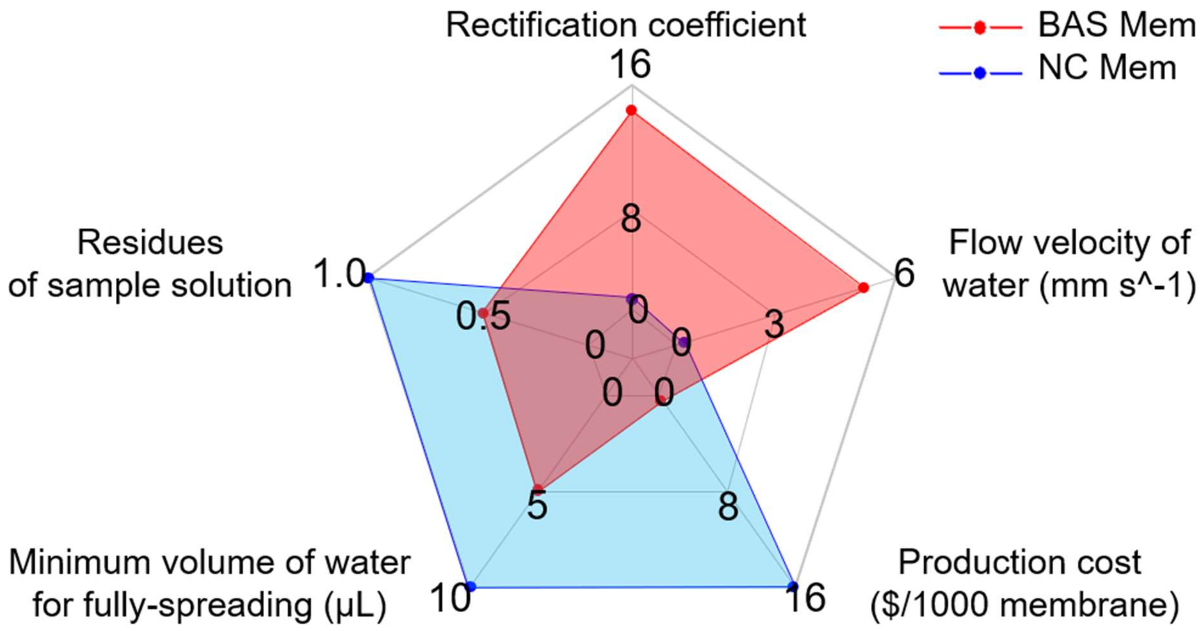

Supplementary Fig. 15 | Radar plot shows the performance of BAS Mem and the comparison verse NC Mem when BAS Mem was used as the component of lateral-flow strips.

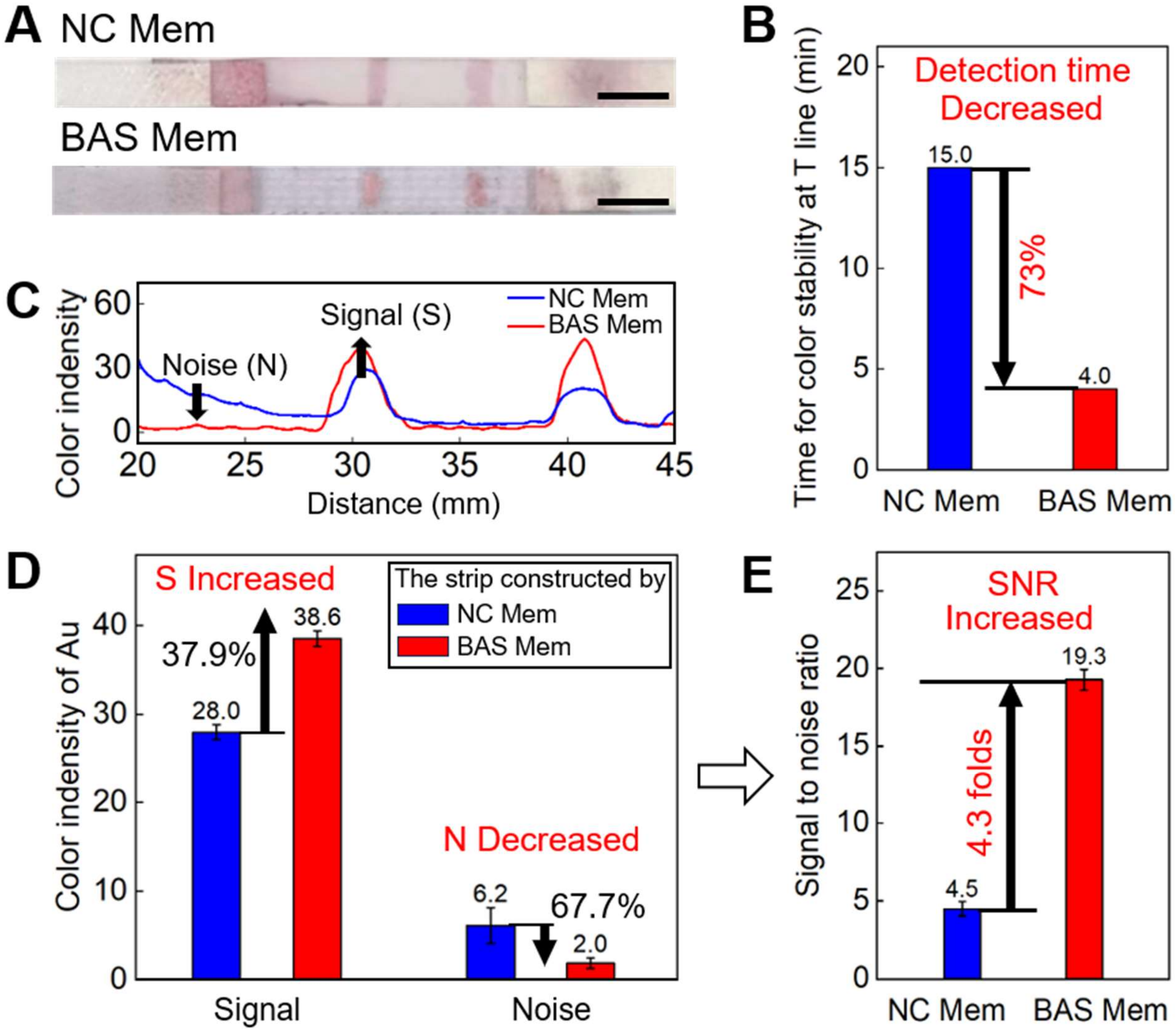

Supplementary Fig. 16 | Results of lateral-flow strips constructed with BAS Mem and NC Mem for the assay of positive sample solution. (**A**) Optical images showing the lateral-flow strips that constructed with NC Mem and BAS Mem, respectively, with visible color on membranes. Scale bar = 5 mm. (**B**) Histogram showing the detection time decreased using the lateral-flow strips constructed by BAS Mem, compared to NC Mem. (**C**) The plot showing the variation of color intensity on the lateral-flow strips constructed with BAS Mem and NC Mem, respectively. (**D**) Histogram showing the increase of signal and the decrease of noise using lateral-flow strips constructed by BAS Mem, compared to NC Mem. (**E**) Histogram showing the increase of SNR by using lateral-flow strips constructed by BAS Mem, compared to NC Mem. The bars in **D** and **E** represent mean ± SD of three independent experiments.

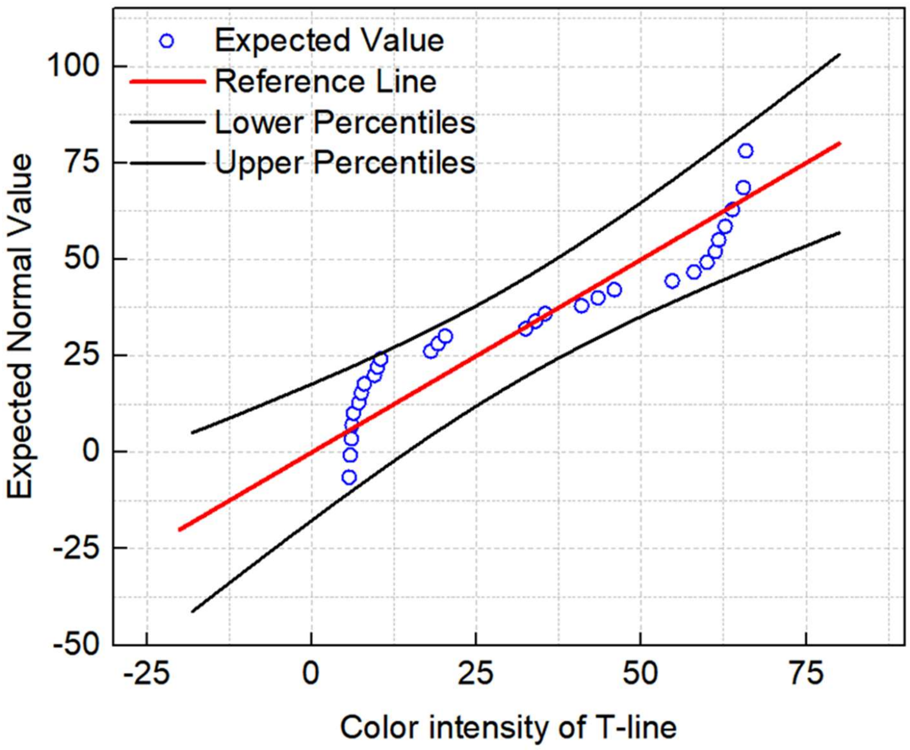

Supplementary Fig. 17 | The Q-Q plot exhibits the results for mimic samples taken from Figure 4.

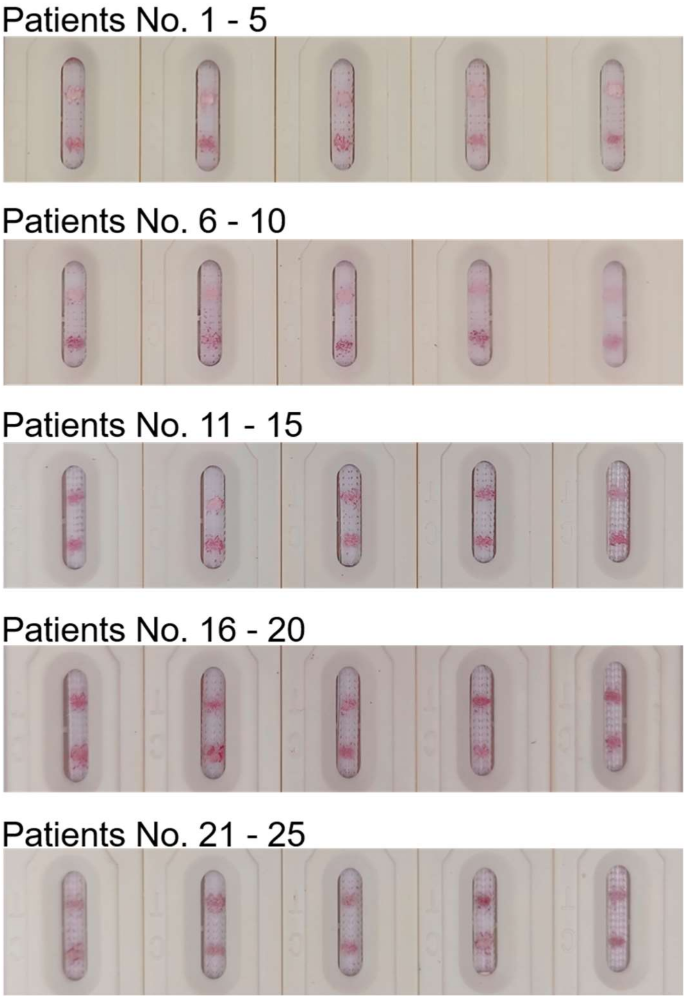

Supplementary Fig. 18 | Images show the original results of 25 serum samples tested by lateral-flow strips.

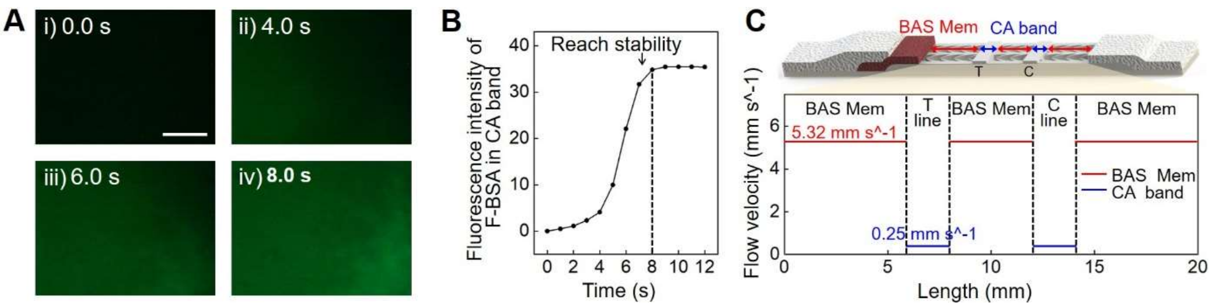

Supplementary Fig. 19 | Characterization of flow velocity of F-BSA solution at the T line, and on the lateral-flow strip. (**A**) Optical images showing the time-dependent flow of F-BSA solution in the CA band. Scale bar = 0.5 mm. (**B**) The plot showing the fluorescence intensity of F-BSA in CA band with time. (**C**) The schematic graph and plot illustrating the flow velocity of liquid is 5.32 mm s^-1 on BAS Mem, but is 0.25 mm s^-1 at the T and C lines (basically referred to the CA band) of the lateral-flow strip.

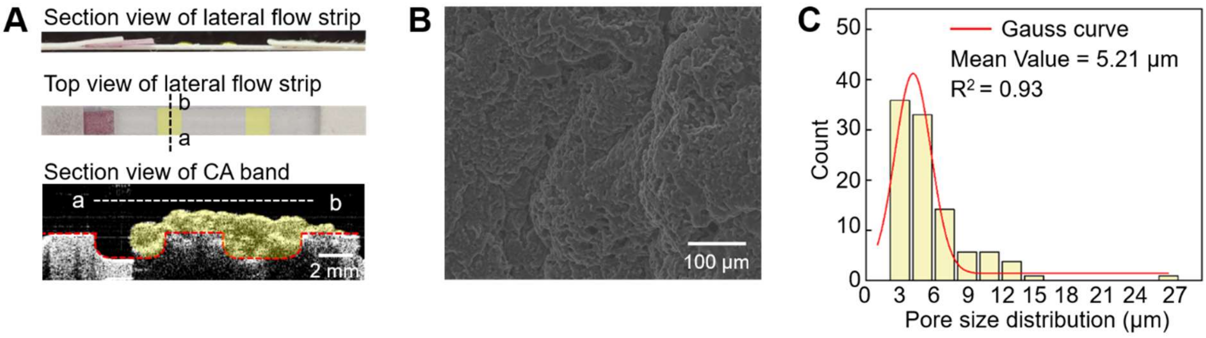

Supplementary Fig. 20 | Characterization of the shape, microstructure, and pore size distribution of CA bands. (**A**) Optical images showing the structure of lateral-flow strips and CA bands. (**B**) SEM image showing the internal structure of the CA bands. (**C**) Histogram of pore size distribution of CA bands.

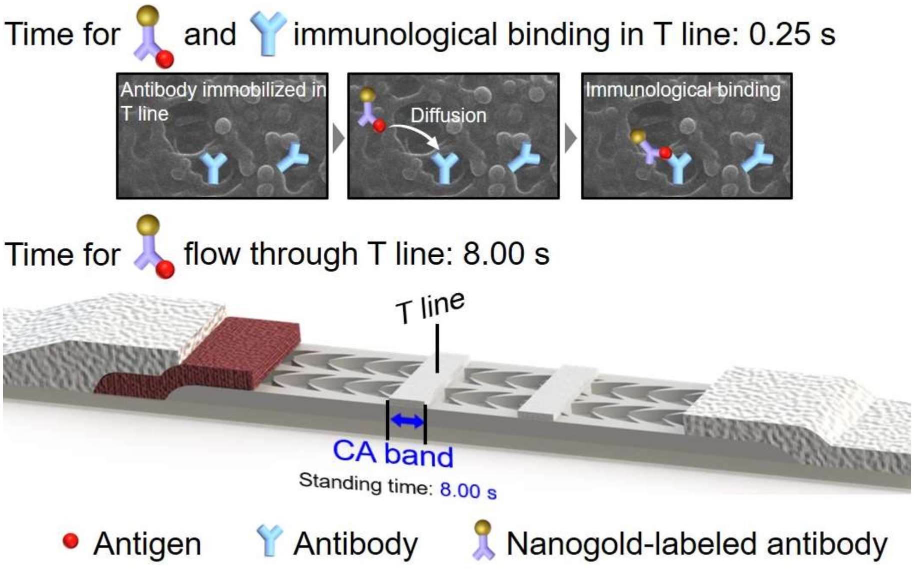

Supplementary Fig. 21 | The schematics shows the time is sufficient for the immunological binding between the "antigen- antibody- nanogold" complex and the antibody immobilized at the T line.

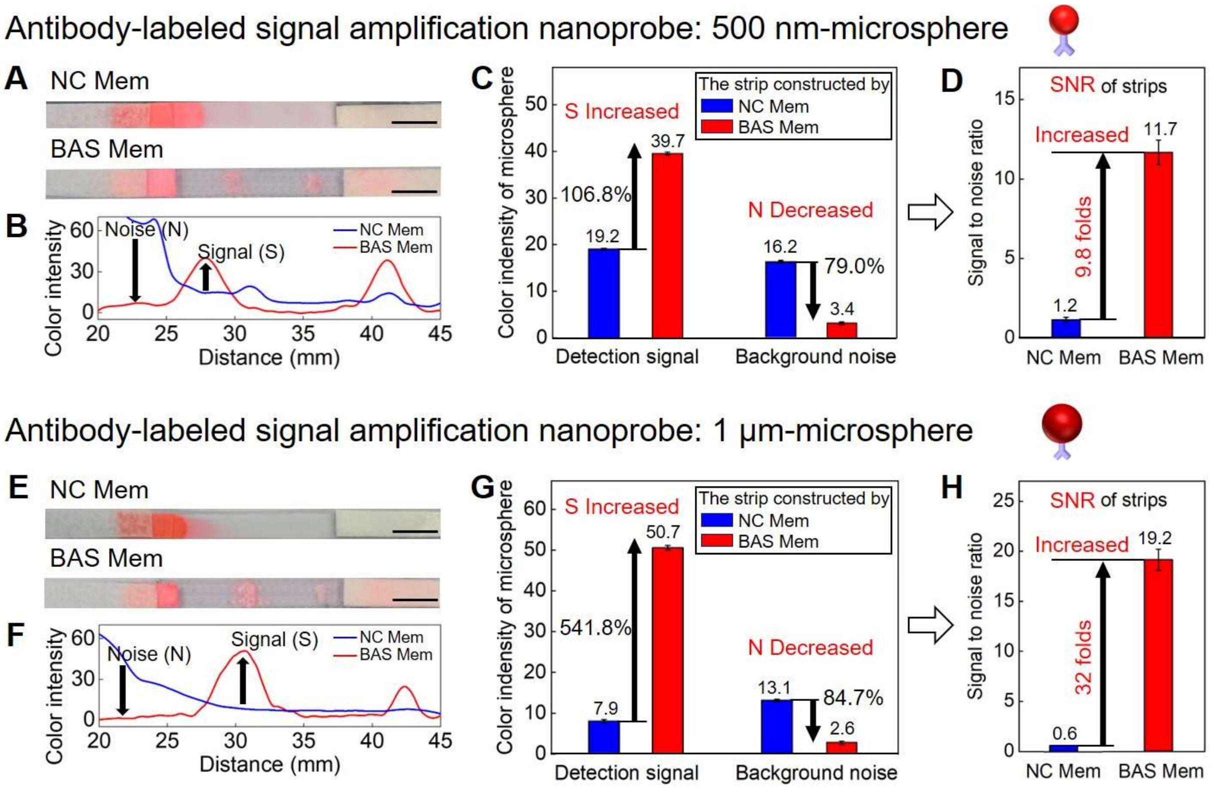

Supplementary Fig. 22 | Results of lateral-flow strips constructed with NC Mem and BAS Mem for the assay of positive sample solution using different types of antibody-labeled signal amplification probe. (**A**)-(**D**) Using 500 nm-gold particle as the antibody-labeled signal amplification nanoprobe. (**E**)-(**H**) Using 1 μm-microsphere as the antibody-labeled signal amplification microprobe. (**A**) and (**E**) Optical images showing the lateral-flow strips that constructed with NC Mem and BAS Mem, respectively, with visible color on membranes. Scale bar = 5 mm. (**B**) and (**F**) The plots showing the variation of color intensity on lateral-flow strips constructed with NC Mem and BAS Mem, respectively. (**C**) and (**G**) Histograms showing the increase of signal and the decrease of noise by using lateral-flow strips constructed by BAS Mem, compared to NC Mem. (**D**) and (**H**) Histograms showing the increase of SNR by using lateral-flow strips constructed by BAS Mem, compared to NC Mem. The bars in **C**, **D**, **G** and **H** represent mean ± SD of three independent experiments.

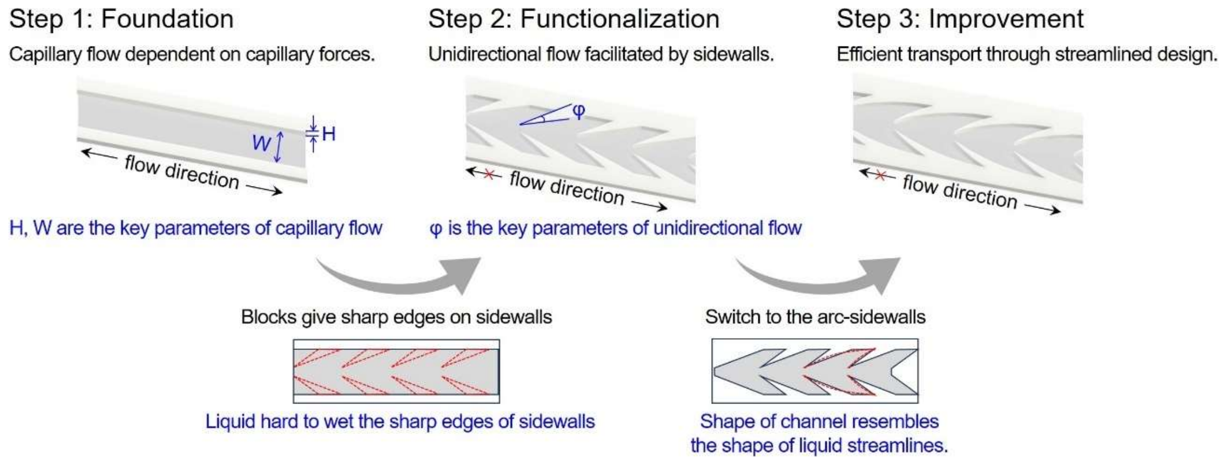

Supplementary Fig. 23 | Schematic diagram showing the design concept of the BAS Mem.

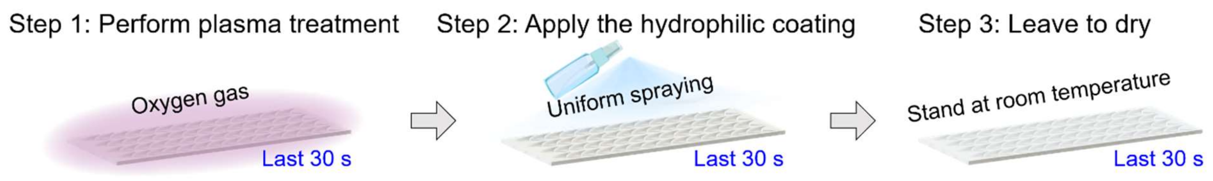

Supplementary Fig. 24 | Schematic graphs showing the steps for preparing the hydrophilized BAS Mem.

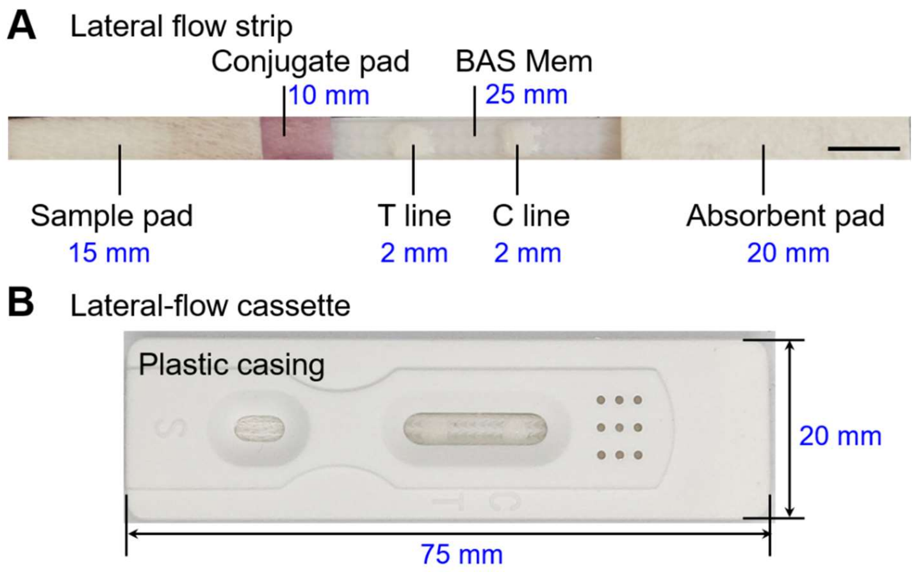

Supplementary Fig. 25 | Characterization of the lateral-flow strip made of BAS Mem. (**A**) Optical image showing the structure of the lateral-flow strip, and the dimensions of each component. (**B**) Optical image showing the physical layout of a lateral-flow cassette.

Supplementary Table 1 | Table exhibits the parameters of membranes with different angles, φ. Here, φ=α-β.

	W(mm)	S(mm)	φ(°)	α(°)	β(°)	H(mm)	L_1(mm)	L_2(mm)
φ -10°	0.16	0.24	10	30	20	0.32	1.00	0.6
φ -15°	0.16	0.24	15	45	30	0.32	1.00	0.6
φ - 25°	0.16	0.24	25	65	40	0.32	1.00	0.6

Supplementary Table 2 | Table exhibits the parameters of membranes with different heights, H.

	W(mm)	S(mm)	φ(°)	α(°)	β(°)	H(mm)	L_1(mm)	L_2(mm)
H -0.08	0.16	0.24	15	45	30	0.08	1.00	0.60
H -0.16	0.16	0.24	15	45	30	0.16	1.00	0.6
H -0.24	0.16	0.24	15	45	30	0.24	1.00	0.6
H -0.32	0.16	0.24	15	45	30	0.32	1.00	0.6
H -0.40	0.16	0.24	15	45	30	0.40	1.00	0.6

Supplementary Table 3 | Table exhibits the parameters of membranes with different scaling factors, W.

	W(mm)	S(mm)	φ(°)	α(°)	β(°)	H(mm)	L_1(mm)	L_2(mm)
W-0.12	0.12	0.18	15	45	30	0.24	0.75	0.45
W-0.16	0.16	0.24	15	45	30	0.32	1.00	0.6
W-0.20	0.20	0.30	15	45	30	0.40	1.25	0.75

Supplementary Table 4 | Table exhibits the parameters of membranes with different spacings, S.

	W(mm)	S(mm)	φ(°)	α(°)	β(°)	H(mm)	L_1(mm)	L_2(mm)
S -0.08	0.16	0.08	15	45	30	0.32	1.00	0.6
S -0.16	0.16	0.16	15	45	30	0.32	1.00	0.6
S -0.24	0.16	0.24	15	45	30	0.32	1.00	0.6
S -0.32	0.16	0.32	15	45	30	0.32	1.00	0.6

Supplementary References

1. Ichikawa, N., Hosokawa, K., & Maeda, R. Interface motion of capillary-driven flow in rectangular microchannel. *J. Colloid Interface Sci.* **280**, 155-164 (2004).
