# Supplementary material for: Barbed arrow-like structure membrane with ultra-high rectification coefficient enables ultra-fast, highly-sensitive lateral-flow assay of cTnI
Source: Nat Commun. 2024 Jul 3;15:5603. doi: 10.1038/s41467-024-49810-z (PMC11222510; doi:10.1038/s41467-024-49810-z)
Supplement: Supplementary file 3 — Description of Additional Supplementary Files [file 41467_2024_49810_MOESM3_ESM.pdf]

Description of Additional Supplementary Files

Supplementary Movie 1: Movie of the flow of liquid on BAS Mem. (Batch No.20)

Supplementary Movie 2: Movie of the flow of liquid on BAS Mem. (Batch No.40)

Supplementary Movie 3: Movie of time-dependent unidirectionally flow of liquid on BAS Mem.

Supplementary Movie 4: High-speed digital movie shows pinning of liquid within a single unit on BAS Mem.

Supplementary Movie 5: High-speed digital movie shows spreading of liquid in a single unit on BAS Mem.

Supplementary Movie 6: Movie shows the flow of liquid on membranes which modified for different contact angles.

Supplementary Movie 7: Movie shows liquid spread freely in all directions on NC Mem.

Supplementary Movie 8: Movie shows unidirectional flow of liquid on BAS Mem.

Supplementary Movie 9: Movie of the flow of liquid on NC Mem.

Supplementary Movie 10: Movie of the flow of liquid on BAS Mem. (Batch No.10)

Supplementary Movie 11: Movie shows the flow of different volumes of liquid on NC Mem.

Supplementary Movie 12: Movie shows the flow of different volumes of liquid on BAS Mem.

Supplementary Movie 13: Movie shows the flow of sample solution of Au-Ab on NC Mem.

Supplementary Movie 14: Movie shows the flow of sample solution of Au-Ab on BAS Mem.

Supplementary Movie 15: Movie shows the assay of a mimic positive sample with a BAS Mem-based strip.

Supplementary Movie 16: Movie shows the assay of a mimic negative sample with a BAS Mem-based strip.

Supplementary Movie 17: Demonstration shows the assay of cTnI in fingertip blood using lateral-flow strips constructed with BAS Mem when AMI occurs.
