# Supplementary material for: Barbed arrow-like structure membrane with ultra-high rectification coefficient enables ultra-fast, highly-sensitive lateral-flow assay of cTnI
Source: Nat Commun. 2024 Jul 3;15:5603. doi: 10.1038/s41467-024-49810-z (PMC11222510; doi:10.1038/s41467-024-49810-z)
Supplement: Supplementary file 21 — Reporting Summary [file 41467_2024_49810_MOESM21_ESM.pdf]

nature portfolio

Corresponding author(s): DAPR

Last updated by author(s): Jun 7, 2024

Reporting Summary

Nature Portfolio wishes to improve the reproducibility of the work that we publish. This form provides structure for consistency and transparency in reporting. For further information on Nature Portfolio policies, see our Editorial Policies and the Editorial Policy Checklist.

Statistics

For all statistical analyses, confirm that the following items are present in the figure legend, table legend, main text, or Methods section.

n/a	Confirmed	
☐	☒	The exact sample size (*n*) for each experimental group/condition, given as a discrete number and unit of measurement
☐	☒	A statement on whether measurements were taken from distinct samples or whether the same sample was measured repeatedly
☐	☒	The statistical test(s) used AND whether they are one- or two-sided<br>*Only common tests should be described solely by name; describe more complex techniques in the Methods section.*
☒	☐	A description of all covariates tested
☒	☐	A description of any assumptions or corrections, such as tests of normality and adjustment for multiple comparisons
☐	☒	A full description of the statistical parameters including central tendency (e.g. means) or other basic estimates (e.g. regression coefficient) AND variation (e.g. standard deviation) or associated estimates of uncertainty (e.g. confidence intervals)
☒	☐	For null hypothesis testing, the test statistic (e.g. *F*, *t*, *r*) with confidence intervals, effect sizes, degrees of freedom and *P* value noted<br>*Give P values as exact values whenever suitable.*
☒	☐	For Bayesian analysis, information on the choice of priors and Markov chain Monte Carlo settings
☒	☐	For hierarchical and complex designs, identification of the appropriate level for tests and full reporting of outcomes
☒	☐	Estimates of effect sizes (e.g. Cohen's *d*, Pearson's *r*), indicating how they were calculated

Our web collection on statistics for biologists contains articles on many of the points above.

Software and code

Policy information about availability of computer code

Data collection	ImageJ 1.52v Java 1.8.0_112, Pco. camware 4.14.0, NIS elements 4.20.00.
Data analysis	Origin 2021, ImageJ 1.52v Java 1.8.0_112, Adobe illustrator 2022 which is graphing plugin.

For manuscripts utilizing custom algorithms or software that are central to the research but not yet described in published literature, software must be made available to editors and reviewers. We strongly encourage code deposition in a community repository (e.g. GitHub). See the Nature Portfolio guidelines for submitting code & software for further information.

Data

Policy information about availability of data

All manuscripts must include a data availability statement. This statement should provide the following information, where applicable:

- Accession codes, unique identifiers, or web links for publicly available datasets
- A description of any restrictions on data availability
- For clinical datasets or third party data, please ensure that the statement adheres to our policy

The data supporting the findings of the study are included in the main text and supplementary information files. Raw data can be obtained from the corresponding author upon request.

Research involving human participants, their data, or biological material

Policy information about studies with human participants or human data. See also policy information about sex, gender (identity/presentation), and sexual orientation and race, ethnicity and racism.

Reporting on sex and gender	Sex and gender were not considered for the study design.
Reporting on race, ethnicity, or other socially relevant groupings	No race, ethnicity, or other socially relevant groupings were considered for the study design.
Population characteristics	The covariate-relevant population characteristics of human research participants is not required in this work.
Recruitment	Samples were collected from the Eighth Affiliated Hospital, Sun Yat-sen University.<br>Samples were collected based on diagnosis of acute myocardial infarction(AMI). We collected the serum samples from healthy individuals / AMI patients under the same study protocol. Basically, 10 serum samples were obtained from healthy individuals, and 15 serum samples were obtained from AMI patients. There was no perceived bias in the recruitment process.
Ethics oversight	Medical Research Ethics Committee of the Eighth Affiliated Hospital of Sun Yat-sen University.

Note that full information on the approval of the study protocol must also be provided in the manuscript.

Field-specific reporting

Please select the one below that is the best fit for your research. If you are not sure, read the appropriate sections before making your selection.

☒ Life sciences ☐ Behavioural & social sciences ☐ Ecological, evolutionary & environmental sciences

For a reference copy of the document with all sections, see nature.com/documents/nr-reporting-summary-flat.pdf

Life sciences study design

All studies must disclose on these points even when the disclosure is negative.

Sample size	The determination of the sample size was primarily based on the requirements outlined in the "Guidance on the Application of Accreditation Criteria for the Medical Laboratory Quality and Competence in the Field of Clinical Immunology" document, which specifies the use of no fewer than five sample solutions for testing.
Data exclusions	No data were excluded from the analysis.
Replication	Data for each experiment were repeated three times independently.
Randomization	The randomization of samples was not relevant. We only have one experimental group, which was to detect the amount of cTnI in samples.
Blinding	The investigators were blinded during the measurement of antibody from serum sample and during data analysis. For the rest of the experiments, blinding was not relevant because the purpose of the study was to evaluate the performance of lateral-flow assay.

Reporting for specific materials, systems and methods

We require information from authors about some types of materials, experimental systems and methods used in many studies. Here, indicate whether each material, system or method listed is relevant to your study. If you are not sure if a list item applies to your research, read the appropriate section before selecting a response.

Materials & experimental systems

n/a	Involved in the study	
☐	☒	Antibodies
☒	☐	Eukaryotic cell lines
☒	☐	Palaeontology and archaeology
☒	☐	Animals and other organisms
☒	☐	Clinical data
☒	☐	Dual use research of concern
☒	☐	Plants

Methods

n/a	Involved in the study	
☒	☐	ChIP-seq
☒	☐	Flow cytometry
☒	☐	MRI-based neuroimaging

Antibodies

Antibodies used	cTnI antibody (OriGene, catalog number 700.485-3-5, clone name TPC 110) (1 mg mL^-1, purity ≥ 95%)

	cTnI secondary antibody (OriGene, catalog number H003, clone name 3H9) (50 µg mL^-1, purity > 95%)<br>Rabbit anti-human IgG (Biobomei, catalog number KT165) (1 mg mL^-1, purity > 95%)
Validation	All primary antibodies used in this study have been validated by the manufacturer, all information is available on the product website:<br>cTnI antibody (OriGene, catalog number 700.485-3-5, clone name TPC 110): https://www.origene.com.cn/ivd/cardiacmarkers/ctni<br>cTnI secondary antibody (OriGene, catalog number H003, clone name 3H9): https://www.origene.com.cn/ivd/cardiacmarkers/ctni<br>Rabbit anti-human IgG (Biobomei, catalog number KT165): http://www.bomeibio.com/goods.php?id=8212

Plants

Seed stocks	N/A
Novel plant genotypes	N/A
Authentication	N/A
